# ﻿New morphological data on *Kurzia longirostris* (Daday, 1898) (Crustacea, Branchiopoda) from the Congo River Basin

**DOI:** 10.3897/zookeys.1257.141692

**Published:** 2025-10-31

**Authors:** Camila Moreira-Silva, Francisco Diogo Rocha Sousa, Lourdes M. A. Elmoor-Loureiro, Mwapu Isumbisho, Hugo Sarmento, Alberto V. Borges, Gilmar Perbiche-Neves

**Affiliations:** 1 Programa de Pós-graduação em Ciências Biológicas (Zoologia), Universidade Estadual Paulista, Instituto de Biociências, Botucatu, Brazil Universidade Estadual Paulista Botucatu Brazil; 2 Universidade Federal de Jataí, Instituto de Biociências, Laboratório de Taxonomia Animal, Jataí, Brazil Universidade Federal de Jataí Jataí Brazil; 3 Unité d’Enseignement et de Recherche en Hydrobiologie Appliquée, Département de Biologie-Chimie, ISP/Bukavu, Bukavu, Democratic Republic of the Congo Département de Biologie-Chimie, ISP/Bukavu Bukavu Democratic Republic of the Congo; 4 Universidade Federal de São Carlos, Departamento de Hidrologia, São Carlos, Brazil Universidade Federal de São Carlos São Carlos Brazil; 5 University of Liège, Chemical Oceanography Unit, Liège, Belgium University of Liège Liège Belgium

**Keywords:** Africa, Chydoridae, Congo Basin, endemism, taxonomy

## Abstract

Africa is recognized for its high levels of endemism across many groups of organisms, including Cladocera. Several studies contributed to our understanding of the diversity and geographic distribution of some groups of Chydoridae on this continent. The literature, however, points to species presumed to occur naturally on other continents, suggesting that both diversity and endemism in Africa continue to be underestimated. Despite the absence of more comprehensive knowledge about the morphology of *Kurzia longirostris* (Daday, 1898) from the *terra typica* (Oriental region), our findings revealed small morphological differences between populations of the Congo River when compared with literature reports. Looking at the high morphological variability along the range of its geographic distribution, it becomes clear that *K. longirostris* might be indicated as a species complex. Thus, the idea of continental endemism should be tested in a future revision of the group.

## ﻿Introduction

The cladoceran fauna of Africa is recognized for its high endemism, especially regarding Chydoridae (Chiambeng and Dumont 1998; Sinev 2006, 2008; Smirnov 2008; Van Damme and Dumont 2008, 2009; Van Damme and Eggermont 2011; Van Damme et al. 2011, 2013a; Neretina and Sinev 2021). Currently, the diversity on the African continent is better understood thanks to several studies of species groups within *Leydigia* Kurz, 1875, *Acroperus* Baird, 1843, *Anthalona* Van Damme, Sinev & Dumont, 2011, *Coronatella* Dybowsky & Grochowski, 1894, *Nicsmirnovius* Chiambeng & Dumont, 1999, *Matralona* Van Damme & Dumont, 2009, *Alona* Baird, 1843, and *Biapertura* Smirnov, 1971 *emend.* Sinev 2020 (Van Damme et al. 2003, 2011; Van Damme and Dumont 2008, 2009; Kotov 2009; Sinev 2009; Neretina and Kotov 2015; Van Damme 2016). Nevertheless, literature suggests that there are species with a wide range of distribution in the Old World, found throughout the southern Palearctic, Afrotropical, and Oriental zones, such as *Anthalona harti* Van Damme, Sinev & Dumont, 2011 (Van Damme et al. 2011) and *Leberis punctatus* (Daday, 1898) (Neretina and Sinev 2016). At the same time, there are several reports of taxa considered as species complexes, which might harbor separate local species in Africa, for instance, *Prendalona guttata* (Sars, 1862) (also “*Alona*” *guttata*), *Alona intermedia* Sars, 1862 and *Chydorus sphaericus* (O.F. Müller, 1776) (Dumont et al. 1981; Dumont 1981; Van Damme and Eggermont 2011). These reports suggest that chydorid diversity and endemism in Africa is still underestimated (Van Damme and Dumont 2008, 2009).

*Kurzia longirostris* (Daday, 1898) also occurs in the Afrotropical zone, presenting a wide range of distribution including the Oriental zone (its *terra typica*), Neotropical, South Asian, and Australasian regions (Gauthier 1937; Rajapaksa and Fernando 1986; Rey and Saint-Jean 1969; Smirnov 1971; Dumont 1981; Hudec 2000; Padhye and Dumont 2015; Sinev 2016). The discovery history of this taxon began when Eugen von Daday described *Alona longirostris* (Daday, 1898). Later, Sars (1901) reported the presence of this species in Brazil and suggested its transfer to the genus *Pseudoalona* Sars, 1901. The name *Pseudoalona longirostris* was used in the following years (Brehm 1933, 1934; Gauthier 1937), until the revision from Harding (1957) indicating that this species belonged to the genus *Kurzia* Dybowski & Grochowski, 1894, a classification that has been used since then (Smirnov 1971; Hudec 2000; Sinev 2016; Neretina et al. 2017). Besides *K. longirostris*, this genus is composed of four other species. *Kurzia latissima* Kurz, 1874 with a natural distribution in the Palearctic zone despite reports from Africa and the Neotropics (Smirnov 1971; Chiambeng and Dumont 2005). According to Hudec (2000)*Kurzia media* Birge, 1879 should be considered as a valid species, with reports from the Holarctic and Neotropical zones (Fuentes-Reinés et al. 2022; Andrade et al. 2024). *Kurzia brevilabris* Rajapaksa & Fernando, 1986 is distributed in South East Asia; however, it does not co-occur with *K. longirostris* (Rajapaksa and Fernando 1986). Finally, *Kurzia polyspina* Hudec, 2000 was described (Hudec 2000) from the Neotropical zone and is currently known from Mexico to southern Brazil (Elmoor-Loureiro 2002; Elmoor-Loureiro et al. 2023).

In addition to its wide geographic distribution across tropical and subtropical regions, morphological data on *K. longirostris* indicate considerable variation, particularly in the postabdomen, rostrum, and labrum. These observations highlight the need for a comprehensive taxonomic review, with special attention to detailed limb morphology. To address this, we examined African populations of *K. longirostris* collected from rivers and streams of the Congo River Basin, Democratic Republic (DR) of Congo.

## ﻿Materials and methods

### ﻿Morphological analyses

Observations were carried out in binocular stereo microscope, mounted in drops of glycerin on slides and studied under an Olympus BX41 phase contrast microscope to investigate their morphological traits. The presentation of morphological structures follows the suggestions of Van Damme (2016). To enumerate the limb setae, we adopted the homology criteria of Kotov (2000a, 2000b), which exhibited stability when tested in different groups of cladocerans (Kotov et al. 2010). All drawings were made using a *camera lucida* and digitally covered using a graphic tablet (Wacom Intuos^TM^) and Adobe Illustrator 2020.

### ﻿SEM processing

The samples were initially fixed in 2.5% glutaraldehyde in 0.1 M phosphate buffer (pH 7.3) for 4 h, followed by three washes in distilled water (5 min each). They were then post-fixed in immersed in 0.5% osmium tetroxide in distilled water for approximately 30–40 min (Perbiche-Neves et al. 2015).

Subsequently, the material was washed three× in distilled water (10 min each), followed by dehydration through a graded ethanol series, starting at 7.5% progressing to 100%. Finally, samples were dried by critical point drying, mounted on stubs and sputter-coated for scanning electron microscopy analysis.

All processing and acquisition of scanning electron microscopy (SEM, Quanta 200, FEI Company) images were performed at the Electron Microscopy Center of the Botucatu Institute of Biosciences, UNESP, Botucatu Campus, Brazil.

### ﻿Abbreviations of scientific collections

**FDRS** = Personal collection of Francisco Diogo Rocha Sousa.

Abbreviations used in the figures and the text: **en** = endite; **ep** = epipodite; **ex** = exopodite; **gfp** = gnathobasic filter plate; **gn** = gnathobase; **IP** = interpore distance (distance between the anterior and posterior major head pores); **IDL** = inner distal lobe; **il** = inner lobe; **L1** = First limb; **L2** = Second limb; **L3** = Third limb; **L4** = Fourth limb; **ODL** = outer distal lobe; **PP** = postpore distance (distance between the posterior major head pore and the posterior border of the head shield); **s** = sensillum.

## ﻿Results

### ﻿Taxonomy

**Class Branchiopoda Latreille, 1817**

**Order Anomopoda Sars, 1865**

**Family Chydoridae Dybowsky & Grochowski, 1894 *emend.* Frey, 1967**

**Subfamily Aloninae Dybowsky & Grochowski, 1894 *emend.* Frey, 1967**

**Genus *Kurzia* Dybowsky & Grochowski, 1894**

#### ﻿ Kurzia cf. longirostris

Taxon classificationAnimaliaAnomopodaChydoridae

(Daday, 1898)

764F4EB1-E5F8-5DA7-B98B-2E0AE979E01E

Alona longirostris in Daday (1898)
Alona macrohyncha in Daday (1900)
Pseudoalona longirostris in Sars (1901), Brehm (1933, 1934) and Gauthier (1937)

##### Material examined.

• Eight adults parthenogenetic females from the Congo main river channel, Congo River Basin, DR Congo (-0.60979, 17.6667 and -4.02029, 18.21978), material collected between 17.xii.2013 and 06.v.2015 (FDRS0703). • Five adult females from the Kasai River, Congo River, DR Congo (-3.26218, 17.46914 and -3.26218, 19.2611), material collected between 20.iv.2015 and 26.iv.2015 (FDRS0704). • One adult parthenogenetic female from the Itimbiri River, Congo River Basin, DR Congo (2.06387, 22.69562), material collected on 13.vi.2014 (FDRS0705). • One adult parthenogenetic female from the Ikelemba River, Congo River Basin, DR Congo (0.10862, 18.29738), material collected on 19.vi.2014 (FDRS0706). • One adult parthenogenetic female from the Ruki River, Congo River Basin, DR Congo (0.07411, 18.31294), material collected between 20.vi.2014 (FDRS0707). • One adult parthenogenetic female from the Kamatsha River, Congo River Basin, DR Congo (-3.71521, 18.92626), material collected between 25.iv.2015 (FDRS0708).

**Description of parthenogenetic females.**

**Figure 1.**
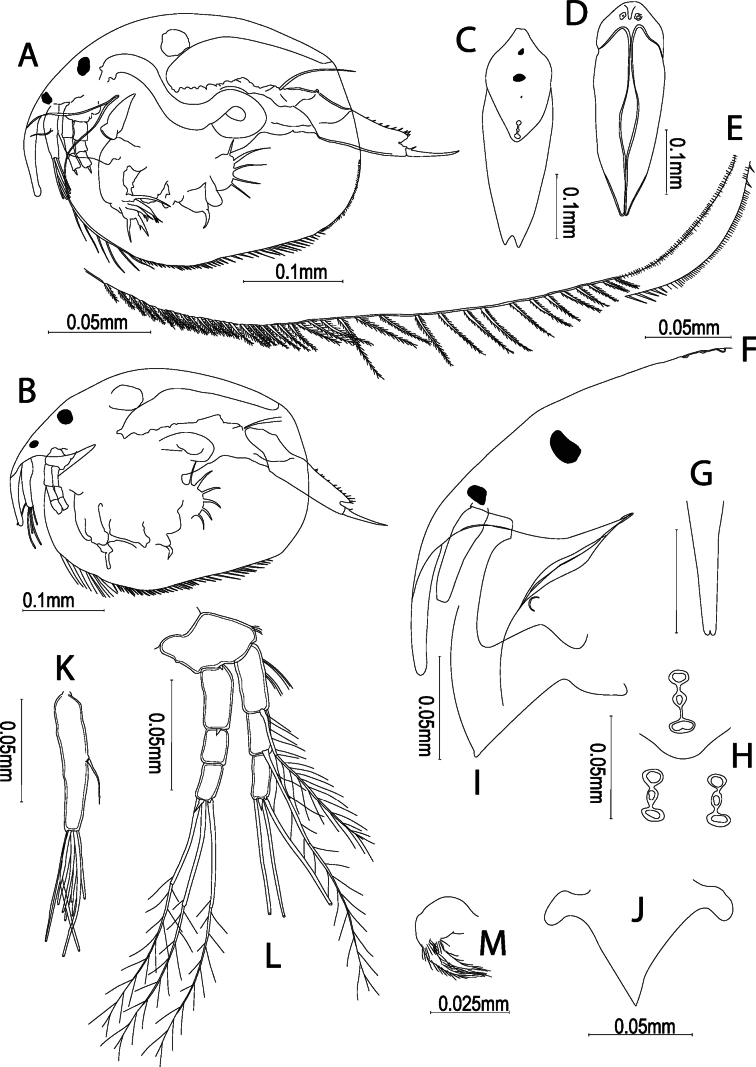
Kurzia cf. longirostris (Daday, 1898) from the Congo River Basin, DR Congo, parthenogenetic female. **A, B.** Habitus; **C.** Dorsal view; **D.** Ventral view; **E.** Ventral margin of carapace, median and posterior portions; **F.** Rostrum; **G.** Idem, frontal view; **H.** Head pores; **I.** Labral keel; **J.** Idem, frontal view showing the lateral horns; **K.** Antennule; **L.** Antenna; **M.** Maxilla.

**General habitus** (Figs 1A, B, 4A, B): rounded body in lateral view, length 0.42–0.52 mm (*n* = 17), height/length ratio 0.68–0.75; dorsal margin arched, with moderate dorsal keel, without lateral projections; in dorsal (Fig. 1C) and ventral views (Fig. 1D); body laterally compressed.

**Carapace** (Figs 1E, 4E): covered by longitudinal lines on valves and head shield; anteroventral margin rounded, with an evident flange; ventral margin almost rounded, with a distinctive rounded angle at 2/3 of margin’s length. Setae at valve ventral margin 38–44, organized into three groups; anterior group with 5 or 6 long setae, median group with up 21 shorter plumose setae, posterior group with up 17 plumose setae. Posterior margin clearly rounded, armed with spinulae exceeding marginal line of valves.

**Cephalic structures** (Figs 1F–J, 4F): ocellus smaller than eye. ***Head shield*** (Fig. 1F) covered by longitudinal lines. ***Rostrum*** (Figs 1F, G, 4C, D) long and slightly curved, in frontal view tip not sharp, about 1.3–2.0× longer than antennular body; posterior margin triangular. ***Head pores*** (Figs 1H, 4G, H): 3 main head pores with anterior and posterior ones longer than median pore, connected by a thick rim; posterior pore transversally elongate, sometimes bilobed; lateral pores inserted in a deep depression, distance from median main head pore about 1.6× PP; PP/IP about 0.42. ***Labrum*** (Fig. 1I, J) short, armed with lateral horns; keel triangular, free of spines or notch, apex round or slightly sharp. ***Antennule*** – A1 (Fig. 1K) approximately 4.5–5.0× longer than wide, never extending beyond tip of rostrum; antennular sensory seta slender, about 2.5–3.1× shorter than length of antennular body, inserted near mid-length of antennular body; 9 aesthetascs which 3 are longer than others but shorter than antennular body, protruding beyond tip of rostrum. ***Antenna*** – A2 (Fig. 1L): basal segment thick, with a short spine; first exopodite segment of similar length to first endopodite segment, armed with 2 clusters of long setulae, apical seta bisegmented and plumose, longer than segment itself; second exopodite segment with a bisegmented, plumose seta equal in length to longest apical setae of third segment; apical spine similar in length to endopodite apical spine; first endopodite segment armed with a spine about 2× shorter than apical spine on third segment; antennal formula (exo/endo): spines 001/101, setae 113/003. ***Maxilla*** (Fig. 1M) well developed, with 2 long setulate setae.

**Figure 2.**
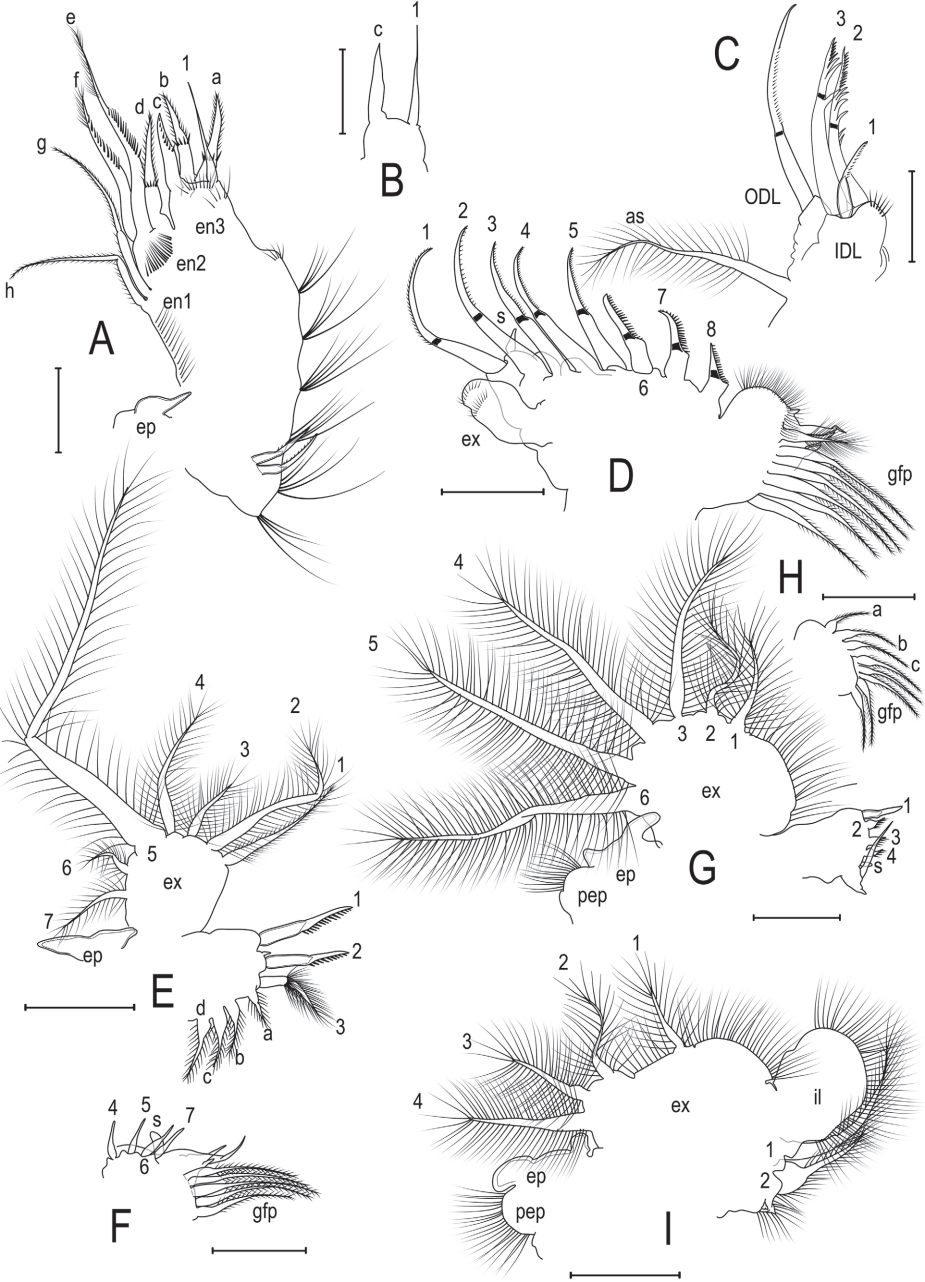
Kurzia cf. longirostris (Daday, 1898) from the Congo River Basin, DR Congo, parthenogenetic female. **A.** First limb; **B.** Idem, endite 3; **C.** Idem, ODL and IDL; **D.** Second limb; **E.** Third limb; **F.** Idem, basal endite; **G.** Fourth limb; **H.** Idem, basal endite and gnathobasic filter plate; **I.** Fifth limb. Scale bars: 0.05 mm.

**Thoracic limbs** (Figs 2A–I, 4I): 5 pairs of thoracic limbs.

***First limb*** (Figs 2A–C, 4J). Epipodite oval, armed with a short digitiform projection. ODL seta bisegmented, armed with fine, short spines, longer than the IDL third seta; accessory seta plumose, similar in length to ODL seta. IDL (en4) with 1 group of short setulae on corm, 3 setae present; seta 1 armed with spines, length about ½ as long as setae 2–3; seta 2 slightly shorter than seta 3; setae 2–3 chitinized and bisegmented, armed with relatively short, thick proximal spines. Endite 3 with 4 setae; anterior seta 1 thin and unarmed about 1.4× longer than posterior seta (c); posterior setae (a–b) of similar length among themselves, armed with spines on the middle part, shorter than anterior seta 1; seta (c) armed laterally with short spines, shorter than the setae (a–b). Endite 2 with 3 posterior setae present (d–f); seta (d) armed with short spines near to middle part, about 1.7× shorter than seta (e); seta (e) long, armed laterally with short spines; seta (f) about 1.2× longer than seta (d) and 1.3× shorter than seta (e). Endite 1 with 2 posterior setae of similar length (g–h), which are bisegmented and densely setulate on distal part. Ejector hooks all similar length and armed with spines; ventral face of the limb with 6–8 clusters of thick setulae. Gnathobase not studied.

***Second limb*** (Fig. 2D). Exopodite without seta, armed with 2 rows of short spinulae. Inner limb portion armed with 8 scrapers; scraper 1 similar in length of scraper 2; a long element present near to scraper 1 base; scrapers 3–4 similar in length, about 0.8 of scraper 1 length; scrapers 5 shorter than the scarper 3–4, about 0.8 length of scraper 1; scrapers 6–7 of similar length, shorter than the scraper 5, about 0.4 length of scraper 1; scraper 8 shorter than scrapers 6–7, about 0.3 length of scraper 1; scraper 6–8 armed with thicker spines than on other scrapers. Proximal portion of the gnathobase setulate, armed with 4 elements; filter plate with 7 setulate setae.

***Third limb*** (Fig. 2E, F). Epipodite oval, with 2 short projections. Exopodite rectangular armed with 5 distal and 2 lateral setae; seventh seta setulate, longer than the sixth, similar in length to third seta; fifth seta geniculated, densely setulate, about 3.3× longer than fourth seta, about 2.5× longer than second seta; fourth seta densely setulate, about 2.2 longer than third seta; second seta plumose, about 3× longer than third seta, about 1.2× longer than first seta; first seta armed laterally with short setulae. Distal endite with three setae (1–3), seta (1–2) scraper-like, seta (3) curved and armed with many setulae bilaterally implanted; 4 plumose posterior setae increasing in length toward to posterior part of the endite (a–d). Basal endite with 4 soft anterior setae 4–7) of similar length. Gnathobase armed with four elements, first being a cylindrical sensillum, second a geniculated and relatively short seta, third and fourth elements naked; filter plate with five plumose setae.

***Fourth limb*** (Fig. 2G, H). Pre-epipodite oval and densely setulate; epipodite oval with two projections. Exopodite wide, with six plumose marginal setae; sixth seta slightly longer than fifth seta; fourth seta about 0.8 of sixth seta length; third seta about 0.6 of sixth seta length; second seta longer than the first seta, about 0.4 of sixth seta length; first seta about 1.8× shorter than the third seta, about 0.3 of sixth seta length; third seta about 1.5× longer than second seta. Distal endite with 4 setae (1–4); seta 1 chitinized; flaming-torch-like setae (3–4) markedly shorter than the seta 1. Basal endite armed with 3 setulate setae which increase in length towards to gnathobase (a–c). Gnathobase with 2 elements, armed with a seta of similar in length to width of endite; filter plate with 5 setae.

***Fifth limb*** (Fig. 2I). Pre-epipodite rounded and densely setulate; epipodite oval, with 2 projections. Exopodite bilobate, armed with 4 plumose setae; first seta about 2× shorter than fourth seta; second and third setae similar in length, about 1.6× longer than first seta; fourth seta about 1.4× longer than second and third setae. Internal lobe wide, rounded and with many setulae; setae 1–2 setulate; seta 1 about 1.6× longer than seta 2. Gnathobase armed with 2 elements, filter plate absent.

**Figure 3.**
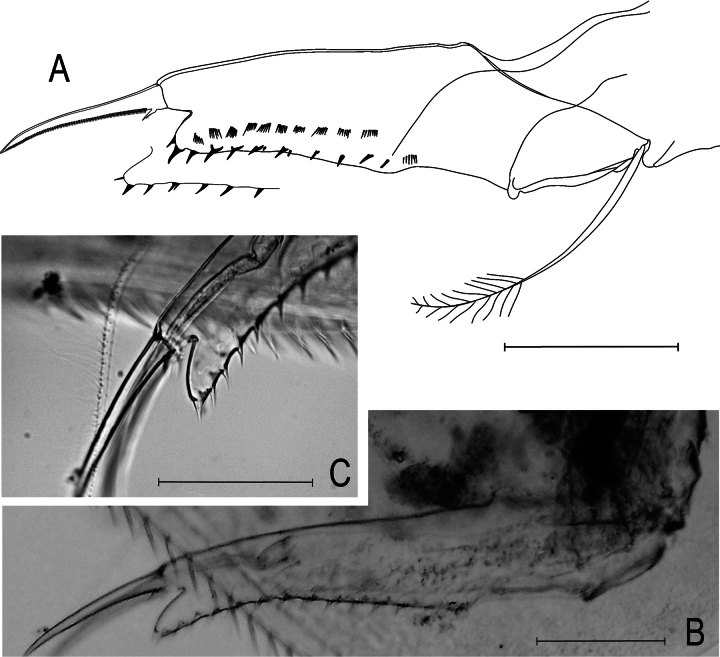
Kurzia cf. longirostris (Daday, 1898) from the Congo River Basin, DR Congo, parthenogenetic female. **A.** Postabdomen; **B.** Idem, illustrating strongly concave postanal dorsal margin; **C.** Idem, detail distalmost part and terminal claws. Scale bars: 0.05 mm.

**Figure 4.**
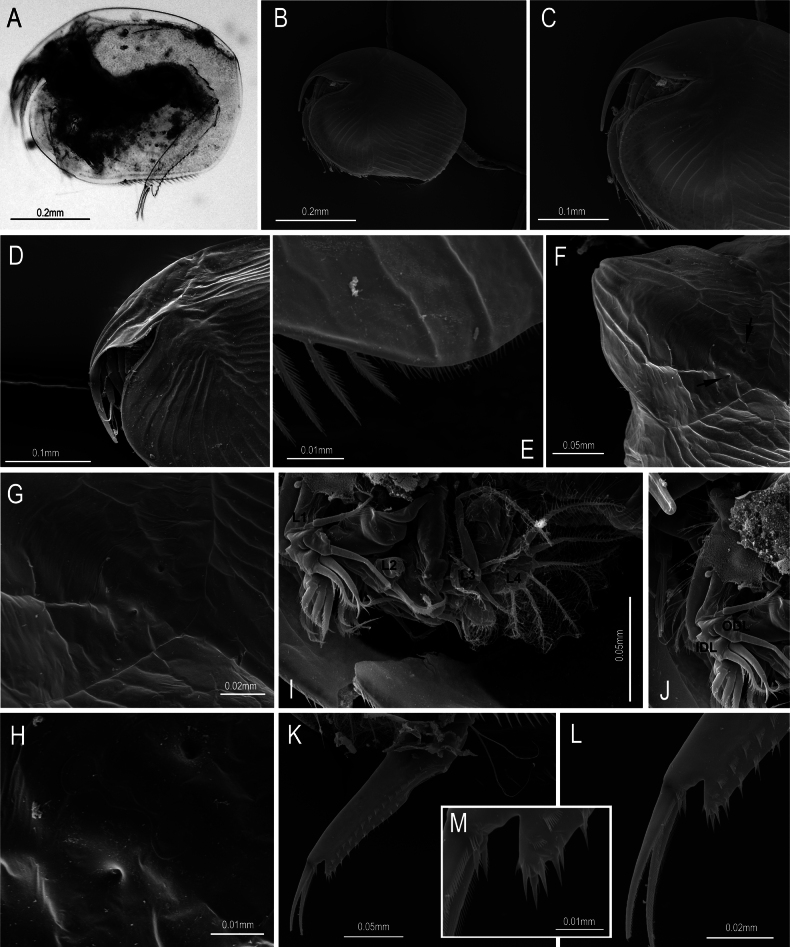
Kurzia cf. longirostris (Daday, 1898) from the Congo River Basin, DR Congo, parthenogenetic female. **A, B.** Habitus; **C, D.** Rostrum; **E.** Posteroventral corner of carapace; **F.** Head shield (arrows show position of lateral head pores); **G, H.** Head pores; **I.** Trunk limbs; **J.** First limb, ODL, and IDL; **K.** Postabdomen; **L.** Idem, postabdominal claws; **M.** Idem, basal spines.

**Abdominal and postabdmominal structures: *Abdomen*** (Fig. 3A). About 3× shorter than thorax, 2 transverse rows of setulae present on dorsal surface. ***Postabdomen*** (Figs 3A–C, 4K–M) narrow, about 4.5–7.5× longer than wide; ventral margin slightly curved; preanal and anal margins of similar length, angles prominent; postanal part elongate, margin markedly concave, distalmost part projected beyond postabdominal claw base; 8–12 marginal denticles, distalmost denticles sometimes isolated, proximal most denticles might be accompanied by 1–4 fine, short spines; 11–16 lateral fascicles formed by thin, short spinulae. Postabdominal setae about 0.6 of postabdomen length, bisegmented, armed with setulae in the distal segment. ***Postabdominal claw*** with spicules on surface, longer than anal margin, about 0.35–0.50 of length of postabdomen; pecten with proximalmost spinulae longer than distalmost ones. ***Basal spines*** (Fig. 4M) armed with spiculae, about 0.08–0.09 of length of postabdominal claw, shorter than width of postabdominal claw at its base.

**Males.** We did not encounter male specimens in our samples. Therefore, males were not studied. However, drawings and a short diagnosis of *K. longirostris* males can be found in Smirnov (1971).

**Ephippial females.** Not studied.

##### Variability.

Two individuals of Kurzia cf. longirostris (Daday, 1898) had two short denticles on the posteroventral corner of the carapace (Fig. 1E). In the postabdomen, the postanal margin might be strongly concave with distalmost part very elongated (Fig. 3B, C). More than concave, mostly the distalmost part was elongated. There were some variations regarding the tip and length of the rostrum.

##### Distribution and biology.

**Figure 5.**
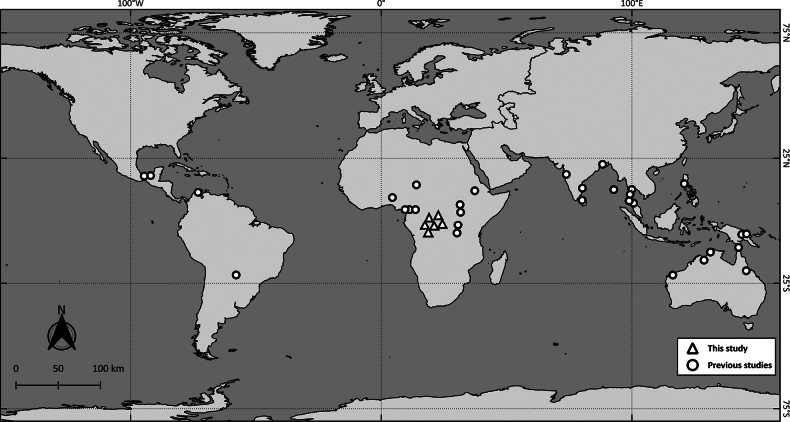
Geographic distribution of *Kurzia longirostris* s.l. (Daday, 1898) based on previous studies (Rey and Saint-Jean 1969; Smirnov 1971; Smirnov and Timms 1983; Rajapaksa and Fernando 1986; Smirnov and De Meester 1996; Hudec 2000; Chiambeng and Dumont 2005; Gutiérrez et al. 2008; Padhye and Dumont 2015; Neretina et al. 2017; Fuentes-Reinés et al. 2022), and GBIF (2025). The new records of Kurzia cf. longirostris (Daday, 1898) from the Congo River Basin, DR Congo, are presented as triangles.

*Kurzia longirostris* s.l. is widely distributed in the Oriental region (*terra typica*) (Daday 1898). In the Australasian region, this species was considered as possibly rare (Smirnov and Timms 1983). Populations from the Neotropical region are observed in a few localities, especially in Brazil and Colombia (Elmoor-Loureiro et al. 2023; Fuentes-Reinés et al. 2022). In the Afrotropics, besides the Congo River Basin (DR Congo), the presence of the species extends throughout the basins of the Volta, Niger, and Chari Rivers (Rey and Saint-Jean 1969; Smirnov 1971; Chiambeng and Dumont 2005; Neretina et al. 2017) (Fig. 5).

The populations examined here were collected from stretches with water temperatures ranging from 25.9 to 28.9 °C, dissolved oxygen range of 1.29–6.76 mg/L^−1^, pH of 3.63–7.18, and conductivity of 13.3–77.1 µS/cm (see Borges et al. 2019 for methods of measuring environmental parameters).

## ﻿Discussion

Hudec (2000) and Elmoor-Loureiro (2002) commented on a potential for *K. longirostris* being a species complex and highlighted the need for a comprehensive revision. *Kurzia longirostris* was initially described from Sri Lanka (Daday 1898), and the complex seems to have a wide distribution in Australasia and Asia (Rajapaksa and Fernando 1986; Hudec 2000; Sinev 2016). Nonetheless, a few studies have also documented presumed populations of *K. longirostris* in the Afrotropical region (Gauthier 1937; Rey and Saint-Jean 1969; Dumont 1981), and its presence there has been attributed to dispersal by birds (Smirnov 1971). This hypothesis should not be discarded, but it needs to be tested by more comprehensive comparison of morphological features between Oriental and African populations.

We suggest that populations studied here differ from *K. longirostris* s.s. (Rajapaksa and Fernando 1986; Hudec 2000). In the African specimens, there are 8–12 marginal denticles on the postabdomen, with 11–16 lateral fascicles, while in *K. longirostris* s.s. there are 10–14 marginal denticles (Rajapaksa and Fernando 1986); however, these characters might be size-dependent. Furthermore, differences are also observed in the proportion of setae on the limbs: anterior seta 1 of the first limb is about 1.4× longer than the posterior seta in African K. cf. longirostris; seta 4 on the exopodite of the third limb is 2.2× longer than seta 3, while they are short and of similar size in *K. longirostris* s.s.; seta 3 on the exopodite of the fourth limb is 1.5× longer than seta 2, and is slightly longer in *K. longirostris* s.s.; seta 1 on the inner face of the fifth limb is about 1.6× longer than seta 2, while it is 2× longer in *K. longirostris* s.s.. In his study of *K. longirostris* populations from the Nile River Basin, Smirnov (1971) illustrated the exopodite of the third and fourth limbs, which are remarkably similar to the material studied here, with differences in the proportion of setae when compared to *K. longirostris* s.s. Nevertheless, these differences require confirmation through a formal redescription of *K. longirostris* from the Oriental region, accompanied by comprehensive morphological documentation, including detailed SEM analyses.

Illustrations of *K. longirostris* populations from several parts of the world suggest consistent morphological variations, especially associated with the postabdomen armature (Sars 1901; Brehm 1933; Gauthier 1937; Rey and Saint-Jean 1969). Several individuals from the African populations studied here also present this type of variation, and the postanal margin of the postabdomen is strongly concave with the distalmost part being very elongate. In some cases, the more concave, the more elongate the distal part was. The posterior main head pore in African populations of K. cf. longirostris may be bilobed in some individuals. Another source of variation was the presence of two short denticles on the posteroventral valve corner. This kind of variation has not been described for any species of *Kurzia* so far. *Kurzia longirostris* in South America (Brazil) bears denticles on the carapace. However, due to the scarcity of material, we could not include it here (Elmoor-Loureiro pers. comm.).

*Kurzia longirostris* should not be regarded as an exceptional case in addressing taxonomic challenges in Africa. Several taxa reported from the continent appear to represent species complexes in need of revision, as already suggested by Van Damme and Maiphae (2013) in their morphological analysis of *Euryalona orientalis* (Daday, 1898) from Southeast Asian populations. Comparable situations have been proposed for *Notoalona freyi* (Van Damme et al. 2013a), *Prendalona guttata* Sars, 1862 (also “*Alona*” *guttata*), *Alona intermedia* Sars, 1862 (Chiambeng et al. 2006; Van Damme and Eggermont 2011), *Chydorus pubescens* Sars, 1901 and *Chydorus eurynotus* Sars, 1901 (Chiambeng and Dumont 2005). The occurrence of these complexes in Central Africa points to considerable cryptic diversity and a strong potential for African endemism. For example, *Alona kolwezii* Van Damme & Dumont, 2008 and *Anthalona simplex* Van Damme, Sinev & Dumont, 2011 may represent endemics restricted to the Congo River Basin (Van Damme and Dumont 2008; Van Damme et al. 2011). Refining the taxonomic resolution of such groups, including *K. longirostris*, is critical for clarifying the true extent of diversity and endemism in Central Africa. At present, the southern region of Africa and high-altitude habitats are recognized as hotspots of chydorid endemism (Van Damme and Eggermont 2011; Van Damme et al. 2013b; Sinev and Dumont 2016; Neretina and Sinev 2021), with the Congo River Basin also emerging as an important center of diversification (Van Damme and Dumont 2008).

In summary, our morphological analysis of African populations of Kurzia cf. longirostris confirms the importance of studying populations from wider subtropical and tropical regions in order to better understand the distribution and diversity in the genus, including the report on *K. media* in Colombia and Brazil (Kotov and Fuentes-Reinés 2015; Andrade et al. 2024) and *K. latissima* in Holarctic and Neotropical regions (Smirnov 1971). Despite the need for a more comprehensive study on the morphology of populations from the Oriental region, it is increasingly clear that *K. longirostris* is a species complex. Thus, the idea of continental endemism (Frey 1987) should be tested in a future revision of the genus *Kurzia*.

## Supplementary Material

XML Treatment for Kurzia cf. longirostris

## ﻿Additional information

### Use of AI

No use of AI was reported.

### Author ORCIDs

Camila Moreira-Silva https://orcid.org/0000-0003-1871-0134

Francisco Diogo Rocha Sousa https://orcid.org/0000-0003-4389-8140

Lourdes M. A. Elmoor-Loureiro https://orcid.org/0000-0001-7439-9753

Hugo Sarmento https://orcid.org/0000-0001-5220-7992

Alberto V. Borges https://orcid.org/0000-0002-5434-2247

Gilmar Perbiche-Neves https://orcid.org/0000-0002-5025-2703

### Data availability

All of the data that support the findings of this study are available in the main text.
